# Complete Genome Sequence of *Ichthyobacterium seriolicida* JBKA-6^T^, Isolated from Yellowtail (*Seriola quinqueradiata*) Affected by Bacterial Hemolytic Jaundice

**DOI:** 10.1128/genomeA.01574-16

**Published:** 2017-02-09

**Authors:** Tomokazu Takano, Yoji Nakamura, Tomomasa Matsuyama, Takamitsu Sakai, Yuya Shigenobu, Takuma Sugaya, Motoshige Yasuike, Atushi Fujiwara, Hidehiro Kondo, Ikuo Hirono, Yutaka Fukuda, Chihaya Nakayasu

**Affiliations:** a National Research Institute of Aquaculture, Japan Fisheries Research and Education Agency, Mie, Japan; b National Research Institute of Fisheries Science, Japan Fisheries Research and Education Agency, Kanagawa, Japan; c Laboratory of Genome Science, Tokyo University of Marine Science & Technology, Tokyo, Japan; d Fisheries Research Division, Oita Prefectural Agriculture, Forestry and Fisheries Research Center, Oita, Japan

## Abstract

*Ichthyobacterium seriolicida* is a fish bacterial pathogen that causes hemolytic jaundice in farmed yellowtail in Japan. To understand more about the characteristics of this bacterium, we determined its complete genome sequence. Two hemolysin genes which may be important for its pathogenicity were identified in the *I. seriolicida* genome.

## GENOME ANNOUNCEMENT

*Ichthyobacterium seriolicida* is a fish bacterial pathogen that causes hemolytic jaundice of yellowtail (*Seriola quinqueradiata*) (1–3). Hemolytic jaundice is one of the most important diseases in cultured yellowtail in Japan, and mortality rates of 5 to 20% are commonly observed (1). *I. seriolicida* is a Gram-negative, aerobic, nonpigmented, long, and curved-rod bacterium that occurs as single cells. The cells show gliding motility but have no pili or flagella and do not form spores (1, 4). Molecular phylogenetic analysis using 16S rRNA and *gyrB* genes revealed that *I. seriolicida* belonged a novel family *Ichthyobacteriaceae* in the order *Flavobacteriales*, class *Flavobacteriia*, phylum* Bacteroidetes*. This bacterium is the sole recognized species in this family (4). To understand more about the characteristics of this unique bacterium at the molecular level, we determined its complete genome sequence.

Genomic DNA of *I. seriolicida* strain JBKA-6^T^ (= ATCC BAA-2465^T^ = JCM 18228^T^) was extracted using an illustra bacterial genomicPrep mini spin kit. We performed *de novo* genome sequencing of strain JBKA-6^T^ using the 454 GS FLX Titanium and Illumina HiSeq 2000 sequencing platforms. A total of 149,635,562 bp (381,492 reads), corresponding to 78-fold coverage of the genome, were obtained from the 454 GS FLX Titanium sequencing. An Illumina mate-paired library was constructed from 3 to 5 kbp of the fragmented genomic DNA and sequenced on a HiSeq 2000 platform to yield a total of 35,819,418,912 bp (356,647,712 reads). Because the HiSeq data were huge (18,600-fold coverage of the genome), we reduced the number of reads by quality filtering (average QV score, ≥30) and random sampling so that the coverage was about 50-fold. The 454 reads and Illumina mate-paired reads were then assembled into seven scaffolds using the Newbler assembler. Sequence gaps between scaffolds and contigs were closed by primer walking using a BigDye Terminator version 3.1 cycle sequencing kit in an ABI 3130 xl genetic analyzer. Genome annotation was performed on the Rapid Annotations using Systems Technology (RAST) server (5).

The genome of *I. seriolicida* strain JBKA-6^T^ is a single circular chromosome (1,921,407 bp; 32.8% G+C content), which contained 1,474 coding DNA sequences (CDSs), one rRNA operon, and 32 tRNA sequences. Among the 1,474 CDSs, a total of 486 genes were annotated as hypothetical proteins. Among the known genes, 131 were categorized as being associated with either DNA or RNA metabolism, 121 as protein metabolism, 95 as cofactors, vitamins, prosthetic groups, and pigments, and 89 as carbohydrates. In addition, 24 genes were predicted to be associated with virulence, disease, and defense. It has been reported that genes involved in gliding motility and the type IX secretion system, such as *gld* and *spr*, are common in the phylum *Bacteroidetes* (6). These genes were also conserved in the genome of strain JBKA-6^T^ and thus may contribute to the phenotypic traits of *I. seriolicida*. Two hemolysin genes that shared 49.3% identity at the nucleotide level were identified in the genome of strain JBKA-6^T^. These hemolysin genes may have crucial roles in the hemolytic activity and pathogenicity of *I. seriolicida*.

### Accession number(s).

The complete genome sequence of *Ichthyobacterium seriolicida* JBKA-6^T^ has been deposited in DDBJ/EMBL/GenBank under accession no. AP014564.
